# Anti-inflammatory activities of puerarin in high-fat diet-fed rats with streptozotocin-induced gestational diabetes mellitus

**DOI:** 10.1007/s11033-020-05816-6

**Published:** 2020-09-18

**Authors:** Wenting Xu, Mengyu Tang, Jiahui Wang, Lihong Wang

**Affiliations:** Department of Reproduction, Zhangjiagang TCM Hospital Affiliated to Nanjing University of Chinese Medicine, Zhang jiagang, Suzhou, Jiangsu China

**Keywords:** Puerarin, Gestational diabetes mellitus (GDM), Signalling pathway, IRS-1

## Abstract

To investigate the effect of puerarin on insulin resistance and inflammation in rats with gestational diabetes mellitus (GDM). Gestational diabetic model rats were established by intraperitoneal injection of streptozotocin (25 mg/kg) combined with high-fat feeding and were randomly assigned to three groups: the control group, the GDM group, and the puerarin-treated group. Puerarin was intragastrically administered to rats daily until the offspring were born. The rats in both the GDM group and control group were administered the same volume of normal saline. Serum total cholesterol, triglycerides, high-density lipoprotein cholesterol, and low-density lipoprotein cholesterol in all groups of rats were measured. Haematoxylin and eosin staining was used to evaluate morphological changes in the liver, pancreas, and adipose tissues around the reproductive organs. Western blotting was carried out to measure the protein expression of IRS-1 and inflammatory factors, including TNF-α, TLR4, MyD88 and phosphorylated NF-κB, in the adipose tissues around the reproductive organs. Puerarin had preventive effects on GDM-induced pathological changes and ameliorated glucose and lipid metabolism disorders in GDM rats. Puerarin upregulated IRS-1 expression and decreased the protein expression of TNF-α, TLR4, and MyD88 as well as the levels of phosphorylated NF-κB in adipose tissues around the reproductive organs in GDM rats. This study indicated that puerarin exerts anti-inflammatory effects by downregulating the important TLR4/MyD88/NF-κB inflammatory signalling pathway. Therefore, puerarin can decrease the expression of TNF-α and ameliorate insulin resistance in GDM rats, suggesting the potential efficacy of puerarin in GDM treatment.

## Introduction

Gestational diabetes mellitus (GDM) refers to hyperglycaemia in pregnant women with no prior history of diabetes mellitus. It has been reported that the morbidity of GDM is 5–10% and has increased significantly in recent years [1]. Studies reported that GDM affects 3–21.2% of all pregnancies in Asian women [2], and the total incidence of GDM in mainland China was 14.8%, which indicated that China might have the largest number of GDM patients around the world (IADPSG criteria) [3].

Although the pathogenesis of GDM has not been fully clarified, GWAS has provided extensive support for the relationship of genetic polymorphisms with GDM susceptibility, such as MTNR1B polymorphisms rs1387153 and rs10830963 [4]. It is generally believed that GDM is associated with insulin resistance (IR) and high risks to the health of the mother and infant, including high blood pressure, foetal abnormalities, weight problems, and type II diabetes. These findings emphasize the need to optimize therapeutic approaches for GDM to maintain the health of mothers and offspring.

Accumulating evidence suggests that chronic inflammation in GDM patients may cause abnormal regulation of insulin signalling and that inflammatory cytokines play important roles in the development of IR in GDM [5]. Tyrosine phosphorylation of insulin receptor substrate 1 (IRS-1) allows the activation and binding of phosphatidylinositol-3kinase (PI3kinase) in insulin signalling, which leads to insulin’s anabolic actions [6]. TNF-α is a common inflammatory cytokine that may promote IRS-1 serine phosphorylation and downregulate the tyrosine phosphorylation of IRS-1 [7]. Some preclinical studies have demonstrated that toll-like receptors are involved in the occurrence of inflammatory reactions and play an important role in the development of inflammation by mediating the activation of nuclear factor-κB (NF-κB) through the Myeloid differentiation primary response gene 88 (MyD88) pathway [8, 9]. Additionally, the role of toll-like receptor 4 (TLR4) and NF-κB in the development of GDM was explored [10, 11]. We found increased protein expression of TLR4 and excessive inflammatory cytokines in the GDM rat model compared with control animals.

Puerarin is an isoflavonoid extracted from the root of *Puerarin lobata* which has been used in traditional Chinese medicine for thousands of years. The hydrodistillation extraction, high-speed counter-current chromatography, ionic liquid extraction, gas-assisted three-liquid-phase extraction method have been exploited as the general techniques for separating this active compound from herb extract [12]. Commercial puerarin is available from many companies. Over the past decades, evidence suggests that puerarin has therapeutic effects on diabetes mellitus, arteriosclerosis, and myocardial ischaemia in rats as well as anti-inflammatory and hypoglycaemic effects [13–15]. A large number of studies on the pharmacological effects of puerarin have shown that puerarin can improve glucose tolerance and insulin sensitivity in the animal model of type 2 diabetes mellitus (T2DM) [16]. In vitro study also suggested that puerarin protects pancreatic β-cell function by mediating PI3K/Akt pathway [17]. Furthermore, puerarin suppressed the expression of intercellular cell adhesion molecule-1 (ICAM-1) and the activation of NF-κB induced by TNF-α, and prevented the adhesion of TNF-α-stimulated monocytes to endothelial cells. Puerarin could play hypoglycaemic and hypolipidaemic roles in experimental and clinical studies [18]. Our previous study also showed that puerarin has clinical effects on IR and regulates insulin signal transduction [19]. To sum up, the effects of puerarin in type 2 diabetes have been studied deeply now, however, there are very few in-depth studies in the effects of puerarin in gestational diabetes around the world. Only a few Chinese scholars reported the protection of puerarin against oxidative stress injury in gestational diabetic rats in the past decade [20–22].

Gestational diabetes and T2DM have the similar pathological characteristics, both of them have an insulin-resistant state related with some chronic inflammation. Some studies have also indicated that TLR4-mediated release of inflammatory cytokines may be a factor leading to increased glucose levels in GDM patients [23]. As the TLR4/MyD88/NF-κB pathway has been suggested to play a critical role in the development of inflammation, we conducted a study to elucidate whether the hypoglycaemic effect of puerarin was mediated through the TLR4/MyD88/NF-κB signal transduction pathway. Therefore, we hypothesized that puerarin exerts anti-inflammatory and hypoglycaemic effects by regulating TLR4 expression. To evaluate this hypothesis, we considered the inflammatory reaction as the cut-in point for the in vivo experiments. We characterised the effects and potential mechanisms by which TLR4 regulates insulin expression in GDM rats and demonstrated that puerarin may regulate downstream signalling proteins of TLR4 associated with inflammation and metabolism. Our findings provide a possible therapeutic target for the treatment of GDM. The purpose of this study was to investigate the effect of puerarin on streptozotocin (STZ)-induced GDM rats and the underlying mechanisms.

## Methods

### Chemicals

Puerarin was purchased from Beijing Solarbio Science & Technology Company Limited (Beijing, China, P9050). STZ was obtained from Sigma Co., Ltd. (St. Louis, MO, USA, V900890).

### Experimental animals

Experimental Wistar rats (females = 40, males = 20) weighing 130–161 g were obtained from Shanghai Slaccas Laboratory Animal Technology Company Limited and were maintained under controlled environmental conditions (22–26 °C, 40–70% humidity, and a 12-h light–dark cycle). They were provided with tap water and a standard lab diet available ad libitum. All the experimental procedures were conducted in strict conformance with the NIH Guideline for the Laboratory Animals’ Use and Care, and the protocol was approved by the Animal Care Committee of Zhangjiagang TCM Hospital Affiliated to Nanjing University of Chinese Medicine.

### Establishment of the GDM rat model

After 1 week of standard diet feeding, the rats were fed a constant 8-week high-fat diet, and all of the rats’ weights were recorded. The rats were then fasted for 12 h. Those with blood glucose levels higher than 6.67 mmol/L were excluded from this study. Vaginal smears were performed every day to determine the rat’s oestrous cycle, and then rats in the oestrous stage were paired with healthy male rats at a ratio of 2:1. On the following morning, the appearance of sperm or a mucus plug observed by using microscopy suggested that the rat was pregnant for 0 d. The pregnant rats were then labelled and separated from others. One week after mating, if the rats were not pregnant, they were excluded from the subsequent study. Pregnant rats were given an intraperitoneal injection of 1% streptozotocin at a dosage of 25 mg/kg to induce the GDM model. Rats with a blood glucose concentration of between 6.67 mmol/L and 16.67 mmol/L were used as the GDM model.

### Dietary intake and treatment groups

**Fig. 1:**
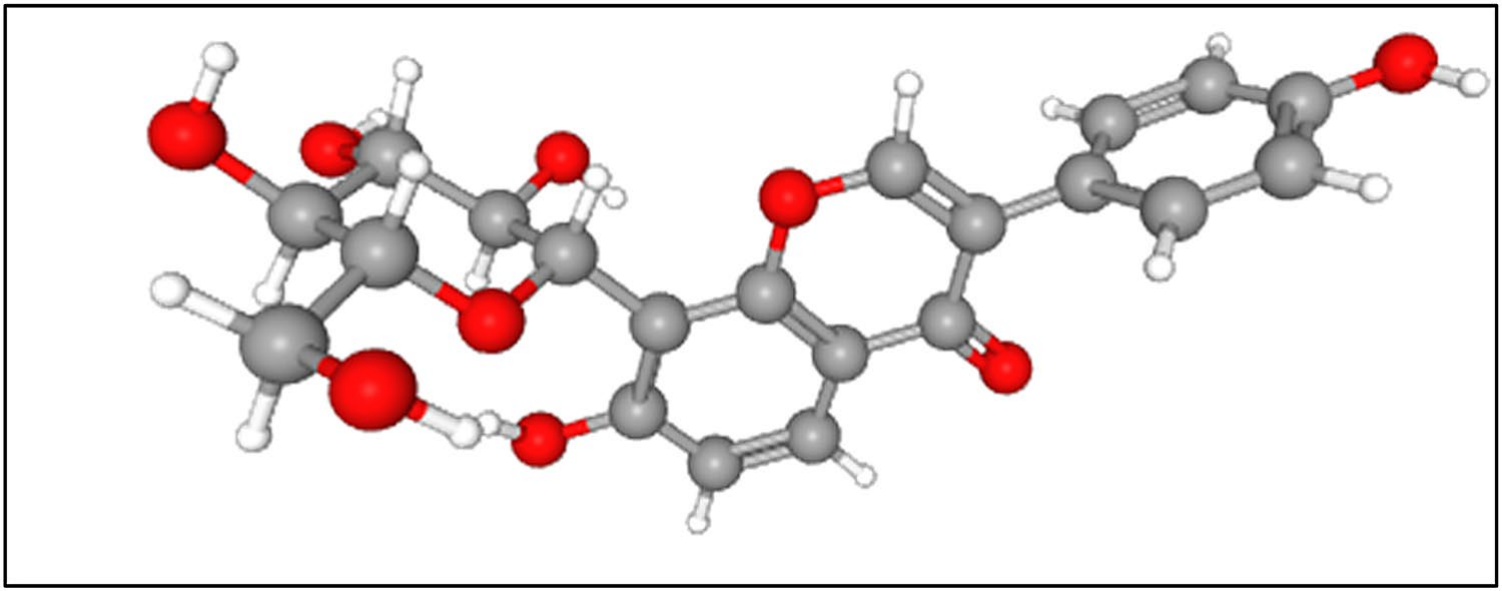
Chemical structure of puerarin

GDM rats were divided into 2 groups (6 rats per group) at random: the GDM group and the puerarin-treated group (resuspended in 0.9% saline, 0.25 g(kg/day), chemical structure shown in Fig. 1). Another 6 healthy, pregnant rats were kept as the control group. The rats were given drug intragastrically every day until their offspring were born. The rats in both the control group and the GDM group were given the same volume of normal saline. All rats were fed the basic diet and provided with water freely. Additionally, all external behavioural and morphological changes, including food and water intake as well as faecal and urinary output, were documented continuously for 2 weeks.

### Collection of blood and tissue samples

Blood samples were gathered at 25 days of pregnancy after fasting for 12 h and animals were euthanized by cervical dislocation. Serum samples were separated after centrifugation of whole blood at 3000 r/min for 10 min, and serum samples were stored in a freezer at − 80 °C until use. The liver, pancreas, and adipose tissues around the reproductive organs were promptly extracted from the rats upon sacrifice. Samples were divided into two portions: one was stored in a 10% formalin solution for histological analyses, and the other was immediately frozen in a freezer at − 80 °C until use.

### Biochemical analyses

Serum total cholesterol (TC), triglycerides (TG), high-density lipoprotein cholesterol (HDL-C), and low-density lipoprotein cholesterol (LDL-C) were assessed using enzymatic kits (eBioscience, San Diego, CA, USA) according to the manufacturer’s instructions. Fast blood glucose levels were measured using instuments as “Bayer fast blood glucose meter”. Insulin levels were measured using Insulin Elisa Kit (Merc& Millipor, St Charles, MO, USA).

### Histological study

Histological analyses of the liver, pancreas, and adipose tissues around the reproductive organs were performed to evaluate morphological changes. Haematoxylin and eosin staining was used.

### Protein extraction and Western blotting

Adipose tissues were homogenized in ice-cold lysis buffer. After homogenization for 30 min on ice, the tissue homogenates were centrifuged at 13,000 r/min for 30 min at 4 °C. The bicinchoninic acid method was applied to determine the total protein concentration. Equivalent amounts of proteins were loaded in each lane, denatured, and then frozen at − 80 °C before western blotting. The proteins were subsequently subjected to sodium dodecyl sulfate polyacrylamide gel electrophoresis and transferred onto polyvinylidene fluoride membranes. After incubation with the apposite primary antibody at 4 °C overnight and secondary antibodies at room temperature for 2 h, polyvinylidene fluoride membranes were washed 3 times in PBS for 10 min, and the targeted proteins were detected by using enhanced chemifluorescence reagent.

The membranes were incubated with antibodies against TLR4 (ab13556, Abcam), IRS-1 (ab131487, Abcam), MyD88 (ab2064, Abcam), NF-κB (8242S, CST), andphospho-NF-κB (3033, CST). All the samples were analysed in an average of 3 independent tests by using different gels. Bands were quantified by ImageJ.

### Statistical analysis

Statistical analyses were performed by GraphPad Prism version 7.0. All data are presented as the means ± standard deviations (SD) for 6 rats per group. Outcomes were analysed by one-way ANOVA followed by Tukey’s multiple comparisons test. A *P* value below 0.05 was considered statistically significant.

## Results

### Tissue pathological changes in GDM rats

**Fig. 2:**
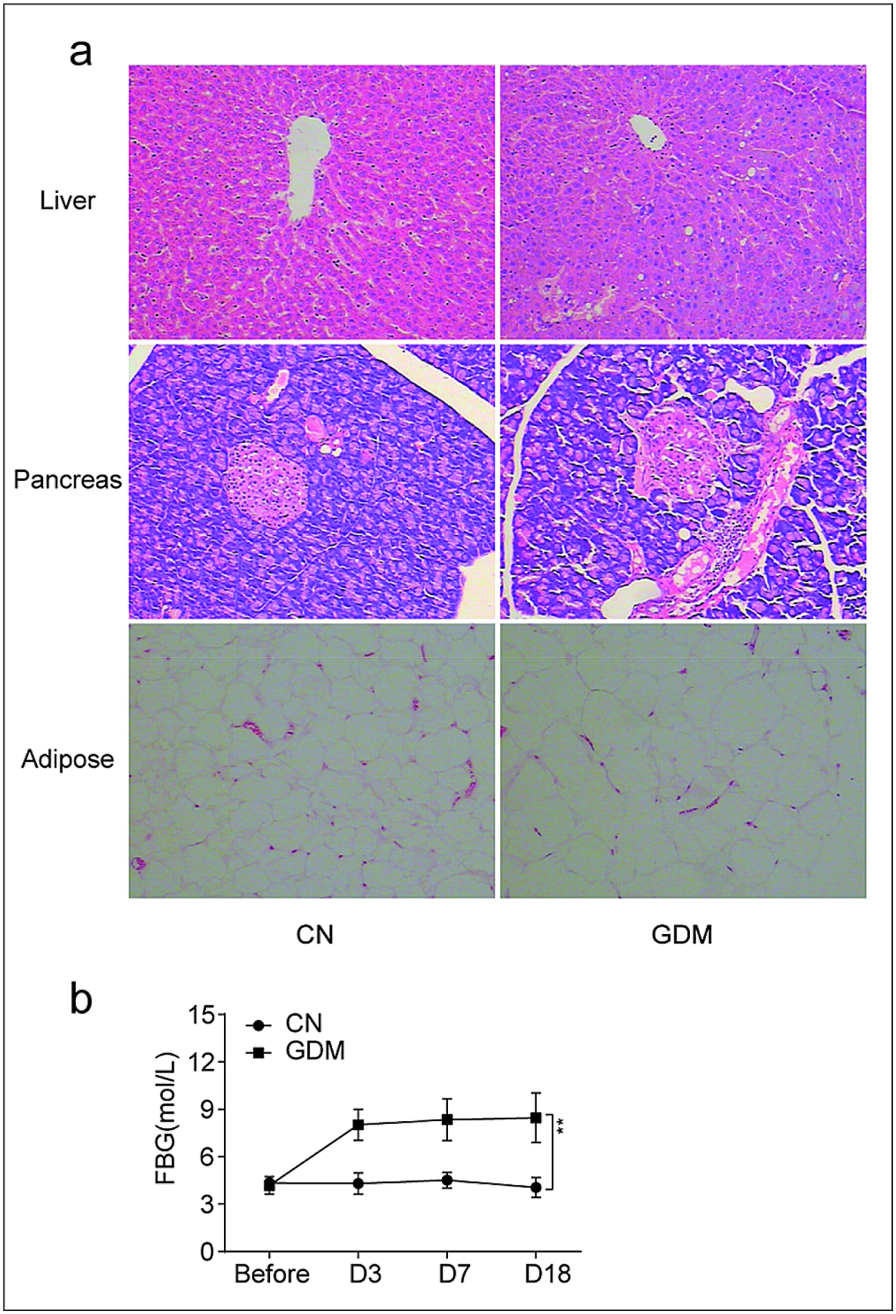
Histopathological observation in GDM rats. **a** Comparison of liver, pancreas, adipose tissues morphology of rats in control group and GDM group (HE, × 100). **b** FBG before and after streptozotocin (STZ) induction in two groups. (*CN* Control Group, *GDM* gestational diabetes mellitus group, *FBG* fasting blood glucose)

The GDM rat model was established with STZ. We used haematoxylin and eosin staining to evaluate the morphology of the liver, pancreas, and adipose tissues in all rats. As presented in Fig. 2a, mild-to-moderate hepatocyte steatosis, focal inflammatory cell infiltration, and individual liver cells undergoing degeneration and necrosis were detected in the GDM group. A morphological study of pancreatic tissues confirmed the mild-to-moderate contraction of pancreatic islets with inflammatory cell infiltration in the GDM group, whereas the fat cells increased in size. To determine the stability of the GDM rat model, after STZ induction, the blood glucose levels on days 3, 7, and 18 were monitored in both groups. The GDM group had higher blood glucose levels than those in the control group, and these levels remained stable over time (P < 0.01) (Fig. 2b).

### Preventive effects of puerarin on GDM-induced pathological changes

**Fig. 3:**
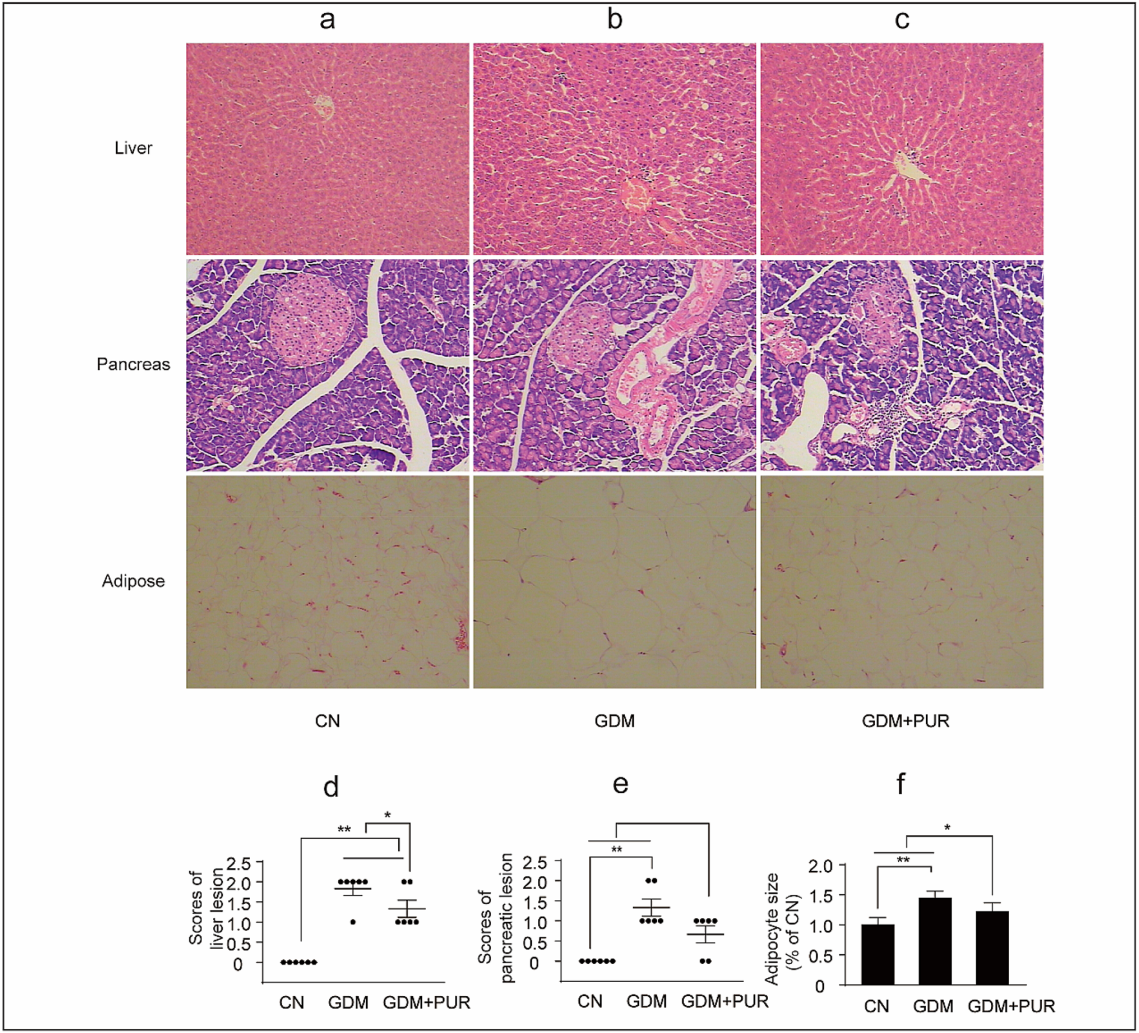
Puerarin administration attenuates GDM rats’ pathological changes. **a** Comparison of liver, pancreas, adipose tissues morphology of rats in control group. **b** Comparison of liver, pancreas, adipose tissues morphology of rats in GDM group. **c** Comparison of liver, pancreas, adipose tissues morphology of rats in puerarin treatment group (HE, × 100). (*CN* Control Group, *GDM* gestational diabetes mellitus group, *GDM + PUR* puerarin treatment group)

Haematoxylin and eosin staining was used to analyse the tissue pathological changes in STZ-induced GDM rats. Pathologist was invited to score the inflammatory changes in pancreatic and liver tissues in each group. (The criteria are shown in the Table 1 and 2). The adipocyte size in the adipose tissue was measured using ImageJ. As shown in Fig. 3a–c, liver cells of the rats in the control group were arranged neatly and no necroinflammatory injury or steatosis and the structure of pancreas were normal. Compared with the control group, the GDM rats showed mild to moderate inflammatory cellular infiltrates and steatosis in liver tissue, pancreatic islet contraction, and an increase in adipocyte size. Puerarin alleviated the degeneration of hepatic cells pancreatic lesion and reduced the adipocyte size (Fig. 3d–f).

**Table 1:** Pancreatic endoscopic diagnostic criteria

Score	Lesion condition of pancreas
0	Normal morphology and structure of pancreas
1	Inflammatory cell infiltration, B cells swelled and occurred vacuolar degeneration; pancreatic acinar cells of some rates occurred vacuolar degeneration, mild interstitial inflammatory cells infiltration; pancreatic ductal revealed mild dilatation
2	Pancreatic B cells particles depigmentation, swelling and occurred vacuolar degeneration, mild atrophy of islet; the number of pancreatic B reduced slightly

**Table 2:** Diagnostic criteria for liver lesion

Score	Lesion condition of Liver
0	Normal hepatocytes arranged in two cell thick plates and no necroinflammatory injury or steatosis
1	Mild inflammatory cellular infiltrates
2	Hydropic degeneration (ballooning) of hepatocytes, focal lytic necrosis, and moderate inflammatory cellular infiltrates
3	Evident fatty change (steatosis)

### Puerarin ameliorated glucose and lipid metabolism disorders in GDM rats

**Fig. 4:**
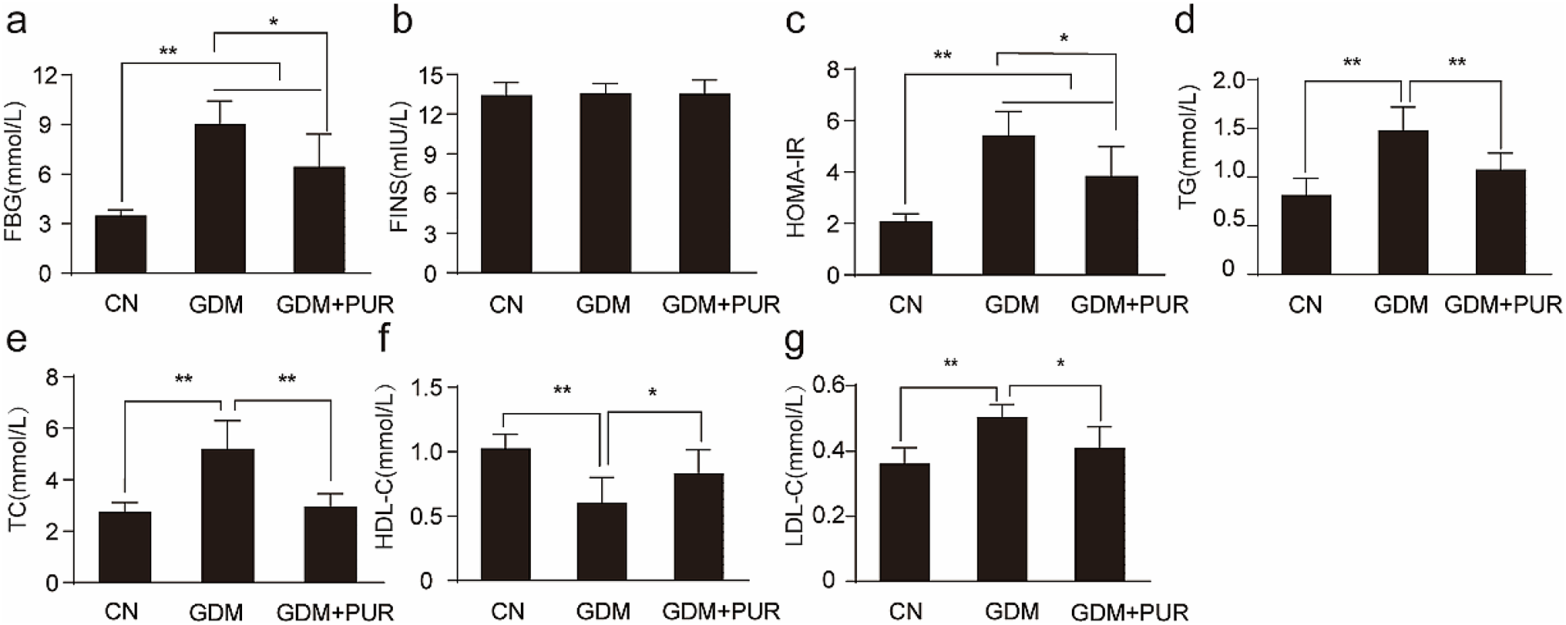
Effects of puerarin on glucose and lipid metabolism in GDM rats. **a** The effect of puerarin on fasting blood glucose (FBG). **b** The effect of puerarin on fasting blood insulin(FINS). **c** The effect of puerarin on HOMA-IR index. **d** The effect of puerarin on triglycerides (TG). **e** The effect of puerarin on total cholesterol (TC). **f** The effect of puerarin on high-density lipoprotein cholesterol (HLDL-C). **g** The effect of puerarin on low-density lipoprotein cholesterol (HDL-C). **P < 0.01, *P < 0.05. (*CN* Control Group, *GDM* gestational diabetes mellitus group, *GDM + PUR* puerarin treatment group)

The effects of puerarin treatment on blood glucose, insulin, TG, TC, HDL-C, and LDL-C are presented in Fig. 4(a–g). Compared with the control group, markedly higher levels of blood glucose, insulin, TG, TC, and LDL-C and lower HDL-C levels were observed in GDM rats. Puerarin treatment effectively ameliorated disorders of glucose and lipid metabolism and IR in GDM rats.

### Effect of Puerarin on TLR4/MyD88/NF-κB signalling molecules

**Fig. 5:**
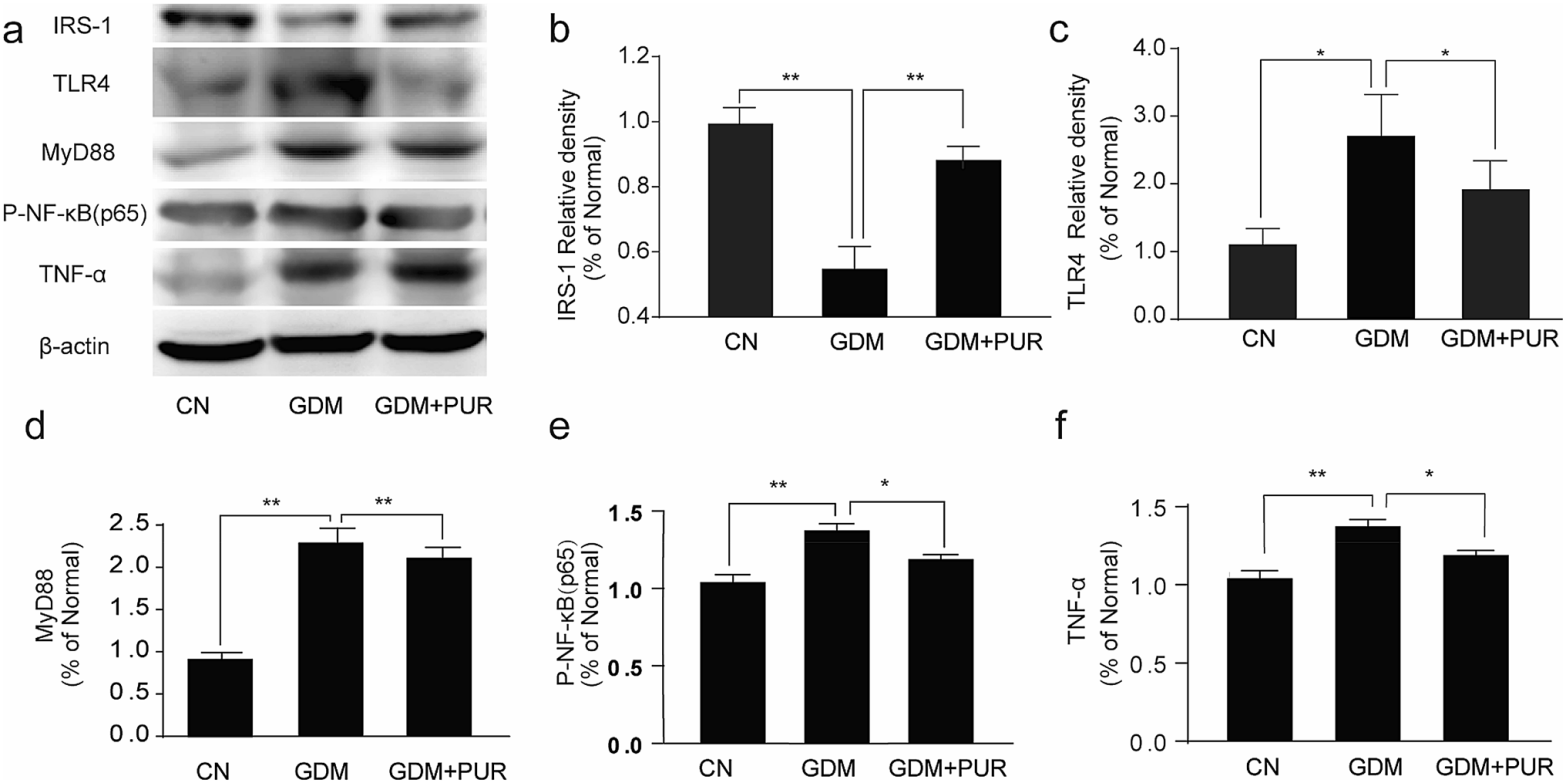
Effects of puerarin on IRS-1, TNF-α and TLR4/MyD88/NF-κB signaling pathway in adipose tissues around the reproductive organs of GDM rats Data are presented as mean ± SEM with (n = 6). **P < 0.01, *P < 0.05

IR is common in the pathogenesis of GDM. The level of IRS-1 was lower in the GDM group than in the control group. Figure 5a–b demonstrates the effects of puerarin on the expression of IRS-1 in GDM rats. Treatment with puerarin upregulated IRS-1 expression in adipose tissues around the reproductive organs in GDM rats (P < 0.01).

To determine the role of the TLR4/MyD88/NF-κB pathway in puerarin-mediated inhibition of the inflammatory response, we studied the activities of TLR4, MyD88, p-NF-κB, and TNF-α in adipose tissues around the reproductive organs in GDM rats by western blotting to elucidate the precise molecular mechanism of the anti-inflammatory activity of puerarin. Based on the western blotting analysis (Fig. 5a, c–d), the protein expression of TLR4 and MyD88 in the adipose tissues around the reproductive organs significantly increased. Puerarin treatment decreased TLR4 and MyD88 expression (P < 0.05). To further confirm the suppression of inflammatory mediators by puerarin, the protein levels of phosphorylated NF-κB and TNF-α in adipose tissues around the reproductive organs were also investigated. As depicted in Fig. 5a, e–f, the expression of phosphorylated NF-κB and TNF-α were enhanced in adipose tissues around the reproductive organs of GDM rats, and puerarin treatment suppressed the expression levels of phosphorylated NF-κB and TNF-α (P < 0.01, P < 0.05).

## Discussion

GDM is characterized by glucose intolerance and IR and is the most common disorder of glucose and lipid metabolism during pregnancy. The aetiology and pathogenesis of GDM is complex and involves genetic and environmental factors, the SNPs study indicated that some genes that have a genetic association in T2DM have the same association in gestational diabetes [24]. GDM can lead to substantial health risks for mothers and offspring [25, 26]. In offspring of women with GDM, there is a high risk of congenital malformations, including heart, digestive system, and nervous system malformations, which can lead to long-term chronic disease [27, 28]. Lifestyle and pharmacological interventions have benefited offspring. Since IR is common in the pathogenesis of GDM, we developed a rat model of GDM by continuously feeding diabetic rats with a high-fat diet and administering an intraperitoneal injection of STZ. We showed that GDM rats had high IR, leading to a larger increase in abnormal lipid metabolism and plasma glucose in comparison with those in normal controls.

Many studies have reported that there is a close correlation between the inflammatory response and insulin resistance [29–31]. Inflammatory cytokines mediate insulin signal transduction by preventing tyrosine phosphorylation of IRS-1 [32, 33]. TLR4 is known to trigger the inflammatory response by activating the NF-κB pathway, which leads to the production of inflammatory mediators [34–36]. Activated TLR4 subsequently transmits inflammatory signals through the adaptor protein MyD88 [37, 38]. The MyD88-dependent signalling pathway activated NF-κB to induce the inflammatory cytokine TNF-α [39]. Recent reports have shown that traditional Chinese medicines have therapeutic potential in inflammatory response and GDM [40–43]. Taking into account the inflammatory response involved in GDM [29, 44], we evaluated the effects of puerarin treatment on IRS-1, TLR4, MyD88, and NF-κB expression in GDM rats. The inhibition of the TLR4/MyD88/NF-κB signalling pathway may highlight the potential of puerarin as a suppressor of the inflammatory response.

In this study, we validated the cross-talk of TLR4 between inflammation and IR in GDM rats. Through its action on inflammation, TLR4 could contribute to the altered inflammation and IR in GDM. This hypothesis is also supported by our present findings showing an association with inflammation signalling molecules, including MyD88, NF-κB, TNF-α, and glucose metabolism.

The Chinese medical herb kudzu root has been used for the treatment of diabetes mellitus for a long time [15, 45, 46], and several previous studies have shown that puerarin has a pharmacological effect of improving glucose uptake and attenuating insulin sensitivity [16]. However, the mechanism by which puerarin affects GDM remains unknown. The present study aimed to investigate puerarin’s effects in GDM rats, and the results strongly suggest that serum glucose and lipid levels were decreased by puerarin in GDM rats. The protein expression levels of IRS-1 were significantly upregulated by puerarin in GDM rats. We also found that puerarin downregulated TLR4 andMyD88 expression and decreased the levels of phosphorylated NF-κB and TNF-α in GDM rat adipose tissues around the reproductive organs.

Natural health products are the potential source in clinical research. Previous studies showed that resveratrol administration can improve maternal glucose and lipid homeostasis in both C57BL/KsJ-Lep ^(db/+)^ (db/+) mouse and streptozotocin-induced diabetes animal model. The toxicity of puerarin to experimental animals is low, studies have reported that there was no significant sub-acute toxicity after administrated intraperitoneally at 516.7 mg/kg and 273.1 mg/kg for 30 days in rats and rabbits observed. However, we should pay more attention to the safety of pregnancy medication. Some study reported that puerarin can pass the placental barrier and maintain a high concentration in fetuses, indicating that puerarin administration should be carefully considered in pregnant women [47]. In this case, the long-term safety of puerarin in animal studies especially in the offspring of the GDM rats need to be determined in our further reaserch.

## Conclusion

In conclusion, our study revealed that puerarin effectively ameliorated disorders of glucose and lipid metabolism and IR in GDM rats and has a potentially anti-inflammatory effect related to the downregulation of downstream pathways of TLR4/MyD88/NF-κB, suggesting that TLR4 is a promising target for the treatment of GDM.

## Abbreviations

GDM: Gestational diabetes mellitus
IR: Insulin resistance
STZ: Streptozotocin
TRL4: Toll-like receptor 4
MyD88: Myeloid differentiation primary response gene 88
NF-κB: Nuclear factor-κB
FBG: Fasting blood glucose
FINS: Fasting blood insulin
TC: Total cholesterol
TG: Triglycerides
HDL-C: High-density lipoprotein cholesterol
LDL-C: Low-density lipoprotein cholesterol
STZ: Streptozotocin

## References

[1] Brawerman GM, Dolinsky VW (2018). Therapies for gestational diabetes and their implications for maternal and offspring health: evidence from human and animal studies. Pharmacol Res.

[2] Chiefari E, Arcidiacono B, Foti D, Brunetti A (2017). Gestational diabetes mellitus: an updated overview. J Endocrinol Invest.

[3] Gao C, Sun X, Lu L, Liu F, Yuan J (2019). Prevalence of gestational diabetes mellitus in mainland China: a systematic review and meta-analysis. J Diabetes Investig.

[4] Alharbi KK, Al-Sulaiman AM, Shedaid KMB, Al-Shangiti AM, Marie M, Al-Sheikh YA, Ali Khan I (2019). MTNR1B genetic polymorphisms as risk factors for gestational diabetes mellitus: a case-control study in a single tertiary care center. Ann Saudi Med.

[5] Khambule L, George JA (2019). The role of inflammation in the development of GDM and the use of markers of inflammation in GDM screening. Adv Exp Med Biol.

[6] Biddinger SB, Kahn CR (2006). From mice to men: insights into the insulin resistance syndromes. Annu Rev Physiol.

[7] Tanti JF, Jager J (2009). Cellular mechanisms of insulin resistance: role of stress-regulated serine kinases and insulin receptor substrates (IRS) serine phosphorylation. Curr Opin Pharmacol.

[8] Choi RY, Ham JR, Lee HI (2017). Scopoletin supplementation ameliorates steatosis and inflammation in diabetic mice.

[9] Verzola D, Bonanni A, Sofia A, Montecucco F, D’Amato E, Cademartori V, Parodi EL, Viazzi F, Venturelli C, Brunori G, Garibotto G (2017) Toll-like receptor 4 signalling mediates inflammation in skeletal muscle of patients with chronic kidney disease. J Cachexia Sarcopenia Muscle 8(1):131–144. 10.1002/jcsm.12129 10.1002/jcsm.12129 PMC5326826 27897392

[10] Li Q, Pereira TJ, Moyce BL, Mahood TH, Doucette CA, Rempel J (1862). Dolinsky VW (2016) In utero exposure to gestational diabetes mellitus conditions TLR4 and TLR2 activated IL-1beta responses in spleen cells from rat offspring. Biochem Biophys Acta.

[11] Kuzmicki M, Telejko B, Wawrusiewicz-Kurylonek N, Lipinska D, Pliszka J, Wilk J, Zielinska A, Skibicka J, Szamatowicz J, Kretowski A, Gorska M (2013). The expression of genes involved in NF-kappaB activation in peripheral blood mononuclear cells of patients with gestational diabetes. Eur J Endocrinol.

[12] Hu W, Shao Q, Xi X, Chu Q, Lan T, Che F, Liu Y, Lu Y, Wei Y (2019). A general gas-assisted three-liquid-phase extraction method for separation and concentration of puerarin, 3’-methoxydaidzin, puerarinxyloside, daidzin and daidzein from puerariae extract. Biomed Chromatogr.

[13] Wang C, Wang W, Jin X, Shen J, Hu W, Jiang T (2016). Puerarin attenuates inflammation and oxidation in mice with collagen antibody-induced arthritis via TLR4/NF-kappaB signaling. Mol Med Rep.

[14] Zhao L, Wang Y, Liu J, Wang K, Guo X, Ji B, Wu W, Zhou F (2016). Protective effects of genistein and puerarin against chronic alcohol-induced liver injury in mice via antioxidant, anti-inflammatory, and anti-apoptotic mechanisms. J Agric Food Chem.

[15] Li W, Zhao W, Wu Q, Lu Y, Shi J, Chen X (2016). Puerarin improves diabetic aorta injury by inhibiting NADPH oxidase-derived oxidative stress in STZ-induced diabetic rats. J Diabetes Res.

[16] Zhou YX, Zhang H, Peng C (2014). Puerarin: a review of pharmacological effects. Phytotherapy Res PTR.

[17] Li Z, Shangguan Z, Liu Y, Wang J, Li X, Yang S, Liu S (2014). Puerarin protects pancreatic beta-cell survival via PI3K/Akt signaling pathway. J Mol Endocrinol.

[18] Jung HW, Kang AN, Kang SY, Park YK, Song MY (2017). The root extract of *Pueraria lobata* and its main compound, puerarin, prevent obesity by increasing the energy metabolism in skeletal muscle. Nutrients.

[19] Wang LH, Wang X, Yu XZ, Xu WT (2016). Potent therapeutic effects of Shouwu jiangqi decoction on polycystic ovary syndrome with insulin resistance in rats. Chin J Integr Med.

[20] Qin JJ, Li RM (2008) Effect of puerarin on insulin resistance in gestational diabetic mellitus rats. Traditional Chinese Drug Research & Clinical Pharmacology

[21] Hui-Bai LI, Wei YC, Liu JM (2015) Protection of puerarin against oxidative stress injury in gestational diabetic rats induced by streptozocin. Drugs & Clinic

[22] Liu J, Immunization DO (2015) Protection effects and mechanism of puerarin on gestational diabetic rats. Pharmacology & Clinics of Chinese Materia Medica

[23] Xie BG, Jin S, Zhu WJ (2014). Expression of toll-like receptor 4 in maternal monocytes of patients with gestational diabetes mellitus. Exp Therap Med.

[24] Khan IA, Jahan P, Hasan Q, Rao P (2019). Genetic confirmation of T2DM meta-analysis variants studied in gestational diabetes mellitus in an Indian population. Diabetes Metab Syndr.

[25] Johns EC, Denison FC, Norman JE, Reynolds RM (2018). Gestational diabetes mellitus: mechanisms, treatment, and complications. Trends Endocrinol Metabol TEM.

[26] Xiang AH, Wang X, Martinez MP, Getahun D, Page KA (2018). Maternal gestational diabetes mellitus, type 1 diabetes, and type 2 diabetes during pregnancy and risk of ADHD in offspring. Diabetes Care.

[27] Persson M, Cnattingius S, Villamor E, Soderling J, Pasternak B, Stephansson O, Neovius M (2017). Risk of major congenital malformations in relation to maternal overweight and obesity severity: cohort study of 12 million singletons. BMJ.

[28] Kaseva N, Vaarasmaki M, Sundvall J, Matinolli HM, Sipola M, Tikanmaki M, Heinonen K, Lano A, Wehkalampi K, Wolke D, Ruokonen A, Andersson S, Jarvelin MR, Raikkonen K, Eriksson JG, Kajantie E (2019). Gestational diabetes, but not pre-pregnancy overweight predicts cardio-metabolic markers in offspring twenty years later. J Clin Endocrinol Metabol.

[29] Feng H, Su R, Song Y, Wang C, Lin L, Ma J, Yang H (2016). Positive correlation between enhanced expression of TLR4/MyD88/NF-kappaB with insulin resistance in placentae of gestational diabetes mellitus. PLoS ONE.

[30] Lee SM, Choi SE, Lee JH, Lee JJ, Jung IR, Lee SJ, Lee KW, Kang Y (2011). Involvement of the TLR4 (Toll-like receptor4) signaling pathway in palmitate-induced INS-1 beta cell death. Mol Cell Biochem.

[31] Shimobayashi M, Albert V, Woelnerhanssen B, Frei IC, Weissenberger D, Meyer-Gerspach AC, Clement N, Moes S, Colombi M, Meier JA, Swierczynska MM, Jeno P, Beglinger C, Peterli R, Hall MN (2018). Insulin resistance causes inflammation in adipose tissue. J Clin Invest.

[32] Hotamisligil GS, Peraldi P, Budavari A, Ellis R, White MF, Spiegelman BM (1996). IRS-1-mediated inhibition of insulin receptor tyrosine kinase activity in TNF-alpha- and obesity-induced insulin resistance. Science (New York, NY).

[33] Paz K, Hemi R, LeRoith D, Karasik A, Elhanany E, Kanety H, Zick Y (1997). A molecular basis for insulin resistance. Elevated serine/threonine phosphorylation of IRS-1 and IRS-2 inhibits their binding to the juxtamembrane region of the insulin receptor and impairs their ability to undergo insulin-induced tyrosine phosphorylation. J Biol Chem.

[34] Jialal I, Kaur H, Devaraj S (2014). Toll-like receptor status in obesity and metabolic syndrome: a translational perspective. J Clin Endocrinol Metab.

[35] Hosseini H, Li Y, Kanellakis P, Tay C, Cao A, Liu E, Peter K, Tipping P, Toh BH, Bobik A, Kyaw T (2016). Toll-like receptor (TLR)4 and MyD88 are essential for atheroprotection by peritoneal B1a B cells. J Am Heart Assoc.

[36] Lucas K, Maes M (2013). Role of the Toll Like receptor (TLR) radical cycle in chronic inflammation: possible treatments targeting the TLR4 pathway. Mol Neurobiol.

[37] Akhter N, Hasan A, Shenouda S, Wilson A, Kochumon S, Ali S, Tuomilehto J, Sindhu S, Ahmad R (2018). TLR4/MyD88 -mediated CCL2 production by lipopolysaccharide (endotoxin): implications for metabolic inflammation. J Diabetes Metabol Disorders.

[38] Miller SI, Ernst RK, Bader MW (2005). LPS, TLR4 and infectious disease diversity. Nat Rev Microbiol.

[39] Akira S, Takeda K, Kaisho T (2001). Toll-like receptors: critical proteins linking innate and acquired immunity. Nat Immunol.

[40] Harikrishnan H, Jantan I (2018). Anti-inflammatory effects of *Phyllanthus amarus* Schum. & Thonn. through inhibition of NF-kappaB, MAPK, and PI3K-Akt signaling pathways in LPS-induced human macrophages. BMC Complement Altern Med.

[41] Chattopadhyay D, Arunachalam G, Mandal AB, Sur TK, Mandal SC, Bhattacharya SK (2002). Antimicrobial and anti-inflammatory activity of folklore: mallotus peltatus leaf extract. J Ethnopharmacol.

[42] Debprasad C, Hemanta M, Paromita B, Durbadal O, Kumar KA, Shanta D, Kumar HP, Tapan C, Ashoke S, Sekhar C (2012). Inhibition of NO(2), PGE(2), TNF-alpha, and iNOS EXpression by *Shorea robusta* L.: an ethnomedicine used for anti-inflammatory and analgesic activity. eCAM.

[43] Wang Y, Feng Q, Niu X, Xu K, Wang Y, Wang J, Li Q (2016). The preventive effect of Zuogui Wan on offspring rats’ impaired glucose tolerance whose mothers had gestational diabetes mellitus. Evid Based Complement Altern Med.

[44] Carrillo-Sepulveda MA, Spitler K, Pandey D, Berkowitz DE, Matsumoto T (2015). Inhibition of TLR4 attenuates vascular dysfunction and oxidative stress in diabetic rats. J Mol Med (Berlin, Germany).

[45] Hou B, Zhao Y, Qiang G (2018). Puerarin mitigates diabetic hepatic steatosis and fibrosis by inhibiting TGF-beta signaling pathway activation in type 2 diabetic rats. Oxid Med Cell Longev.

[46] Chen XF, Wang L, Wu YZ, Song SY, Min HY, Yang Y, He X, Liang Q, Yi L, Wang Y, Gao Q (2018). Effect of puerarin in promoting fatty acid oxidation by increasing mitochondrial oxidative capacity and biogenesis in skeletal muscle in diabetic rats. Nutr Diabetes.

[47] Cao L, Pu J, Cao Q-R, Bo-Wen C (2013). Pharmacokinetics of puerarin in pregnant rats at different stages of gestation after oral administration. Fitoterapia.

